# Changes in the Epidemiology of Multidrug-Resistant Organisms During the COVID-19 Pandemic: A Six-Year Retrospective Study at a Tertiary Care Hospital in Northeastern Thailand

**DOI:** 10.3390/medsci14030366

**Published:** 2026-07-01

**Authors:** Tassawan Pangseeta, Thuksanai Pussadu, Nuntiput Putthanachote, Jaruwan Tawarungruang, Birabongse Hardthakwong, Parichart Boueroy, Ratchadaporn Ungcharoen, Piroon Jenjaroenpun, Anusak Kerdsin, Peechanika Chopjitt

**Affiliations:** 1 Faculty of Public Health, Chalermphrakiat Sakon Nakhon Province Campus, Kasetsart University, Sakon Nakhon 47000, Thailand; tassawan.pa@ku.th (T.P.); jaruwan.t@ku.th (J.T.); birabongse.h@ku.th (B.H.);; 2 Service Quality and Standards Development Division, Roi Et Hospital, Roi-Et 45000, Thailand; thuksanai.ngeng@gmail.com; 3 Clinical Microbiology Laboratory, Roi-Et Hospital, Roi-Et 45000, Thailand; 4 Medical Bioinformatics, Siriraj Genomics, Faculty of Medicine Siriraj Hospital, Mahidol University, Bangkok 10700, Thailand; 5 Siriraj Long-Read Lab (Si-LoL), Medical Bioinformatics Lab, Siriraj Genomics, Faculty of Medicine Siriraj Hospital, Mahidol University, Bangkok 10700, Thailand

**Keywords:** antimicrobial resistance, carbapenem-resistant organisms, COVID-19, hospital-acquired infection, epidemiology, Thailand

## Abstract

**Background:** The COVID-19 pandemic disrupted healthcare systems and antimicrobial stewardship, potentially altering antimicrobial resistance patterns. This study characterized temporal changes in the proportions of multidrug-resistant organisms (MDROs) and identified associated factors before and during the pandemic at a tertiary care hospital in northeastern Thailand. **Methods:** A single-center retrospective observational study was conducted at Roi Et Hospital, including 5458 culture-confirmed MDRO cases (2017–2022), stratified into pre-pandemic (2017–2019) and pandemic (2020–2022) periods. Pathogen-specific resistance proportions were compared using odds ratios (ORs) with 95% confidence intervals (CIs). Multivariable logistic regression identified independently associated factors within each period. **Results:** The proportion of MDRO cases classified as hospital-acquired increased from 40.71% to 57.41% (*p* < 0.001). Carbapenem-resistant *Acinetobacter baumannii* (CRAB) increased markedly (22.87% to 76.11%; OR 10.75, 95% CI 9.43–12.26), followed by carbapenem-resistant *Enterobacterales* (CRE) (4.05% to 21.61%; OR 6.54, 95% CI 5.84–7.32) and carbapenem-resistant *Pseudomonas aeruginosa* (CRPA) (14.32% to 27.15%; OR 2.23, 95% CI 1.87–2.65; all *p* < 0.001). Vancomycin-resistant Enterococcus (VRE) declined significantly (OR 0.41, 95% CI 0.25–0.68; *p* < 0.001). Methicillin-resistant *Staphylococcus aureus* (MRSA) showed a higher proportion among clinical isolates (3.28% to 6.34%; OR 2.01, 95% CI 1.42–2.83), although without a consistent annual trend. In multivariable analyses, ICU admission was independently associated with lower odds of CRE (aOR 0.52) and CRPA (aOR 0.63) and with higher odds of CRAB (aOR 2.13; all *p* < 0.001). **Conclusions:** The COVID-19 pandemic was associated with a major proportional shift toward carbapenem-resistant Gram-negative pathogens, with distinct profiles of associated factors across CRAB, CRE, and CRPA. These findings highlight the need for pathogen-specific infection prevention and antimicrobial stewardship strategies during healthcare system disruptions.

## 1. Introduction

Antimicrobial resistance (AMR) is a major global public health threat that undermines the effective treatment of bacterial infections and is associated with increased morbidity, mortality, and healthcare costs. Recent global estimates indicate that bacterial AMR was associated with 4.71 million deaths in 2021, including 1.14 million deaths directly attributable to resistant pathogens [1]. This burden is expected to be borne disproportionately by low- and middle-income countries, where health systems often face limited diagnostic capacity, constrained infection prevention infrastructure, and high antimicrobial consumption [1,2,3]. In Southeast Asia, AMR is especially concerning because of the high baseline prevalence of resistant pathogens, broad antimicrobial access, and persistent gaps in stewardship and surveillance [4,5,6,7]. In Thailand, AMR imposes substantial clinical and economic consequences. Among the most clinically significant hospital-associated pathogens are carbapenem-resistant Gram-negative organisms, specifically carbapenem-resistant *Acinetobacter baumannii* (CRAB), carbapenem-resistant *Enterobacterales* (CRE), and carbapenem-resistant *Pseudomonas aeruginosa* (CRPA), because of their limited treatment options and poor clinical outcomes [5,8,9,10].

The coronavirus disease 2019 (COVID-19) pandemic placed unprecedented strain on healthcare systems and may have reshaped the epidemiology of multidrug-resistant organisms (MDROs). During this period, hospitals faced surges in critically ill patients, increased use of invasive devices, prolonged hospital stays, workforce shortages, and disruptions to routine infection prevention and control (IPC) and antimicrobial stewardship programs [11,12]. At the same time, empirical antibiotic prescribing increased substantially, with rates exceeding 70% of hospitalized COVID-19 patients in Southeast Asia [4], despite relatively low rates of confirmed bacterial co-infection [13,14,15,16,17]. Together, these conditions created a plausible setting for amplification of healthcare-associated transmission and antimicrobial selection pressure [18].

Published findings have been heterogeneous across pathogens and healthcare settings. Several studies have documented increased incidence and/or proportions of carbapenem-resistant Gram-negative organisms during the pandemic [18,19,20], whereas trends in methicillin-resistant *Staphylococcus aureus* (MRSA) and vancomycin-resistant enterococci (VRE) have been more variable [21,22,23]. This heterogeneity suggests that the pandemic’s effect on AMR depends on local factors, including patient case mix, patterns of antibiotic use [24], hospital organization, and infection prevention and control (IPC) capacity. In Thailand, most published data originate from university-affiliated or metropolitan centers [25], leaving a knowledge gap regarding MDRO epidemiology in provincial tertiary hospitals, which experience unique challenges such as increased patient referral and limited resources during the pandemic [25,26].

Therefore, this study aimed to characterize changes in the proportions of major MDROs and to identify clinical and healthcare-related factors associated with MDRO infection before and during the COVID-19 pandemic in a tertiary care hospital in northeastern Thailand. Specifically, we evaluated temporal changes in the proportions of CRAB, CRE, CRPA, MRSA, and VRE among clinical isolates between 2017 and 2022, and examined factors independently associated with pathogen-specific MDRO infection within each study period. However, longitudinal data describing pathogen-specific AMR dynamics in provincial tertiary hospitals in Thailand remain limited, particularly in the context of COVID-19-related healthcare disruption.

## 2. Materials and Methods

### 2.1. Study Design and Setting

We conducted a single-center retrospective observational epidemiologic study using hospital-based microbiological surveillance and clinical data from Roi Et Hospital, a 1000-bed regional tertiary referral center in northeastern Thailand. The study was reported in accordance with the Strengthening the Reporting of Observational Studies in Epidemiology (STROBE) statement and the STROBE-AMS extension for antimicrobial resistance surveillance [27,28]. Ethical approval was obtained from the Roi Et Hospital Ethics Committee (Approval No. RE068/2023; date of approval: 20 June 2023), which waived the requirement for informed consent because the study used anonymized retrospective data.

### 2.2. Study Population and Study Period

The study included all hospitalized patients with at least one clinical culture positive for target multidrug-resistant organisms between 1 January 2017 and 31 December 2022. The study period was stratified into two phases: the pre-pandemic period (2017–2019) and the pandemic period (2020–2022). For patients with multiple admissions, each admission separated by ≥30 days was counted as a separate case. This 30-day interval was applied uniformly to all specimen types as an operational rule to define distinct infection episodes and to limit duplication. The rule is consistent with the deduplication principles for cumulative susceptibility data described in CLSI guideline M39 and with prior antimicrobial resistance surveillance studies that defined repeat isolates of the same organism within 30 days as the same episode [29,30]. Infection acquisition was classified using CDC/NHSN-based timing criteria, whereby an isolate recovered >48 h after admission and not present or incubating at the time of admission was classified as hospital-acquired, and an isolate identified within the first 48 h was classified as community-acquired (see Section 2.3). This timing-based acquisition classification is conceptually distinct from the hospital stay prior to culture (time at risk) defined in Section 2.4: the former is a binary classification of where the infection was acquired, whereas the latter is a measure of the interval from admission to the first positive culture. A complete history of previous admissions and healthcare exposure (e.g., in the preceding 90 days) was not consistently recorded in the electronic medical record and could therefore not be incorporated.

#### 2.2.1. Inclusion Criteria

Patients were eligible for inclusion if they met all of the following criteria: (1) admitted to an inpatient ward at Roi Et Hospital during the study period; (2) had at least one culture-confirmed MDRO (CRE, CRAB, CRPA, VRE, or MRSA); (3) had a minimum hospital stay of 48 h, regardless of infection acquisition classification; and (4) in cases with multiple positive cultures for the same organism during a single admission, only the first isolate per species per admission was included to improve epidemiologic independence and avoid overestimation of resistance.

#### 2.2.2. Exclusion Criteria

**Figure 1:**
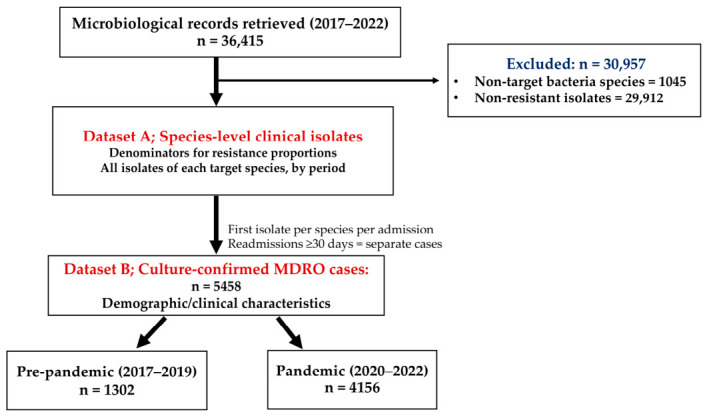
A participant flow diagram illustrating the derivation of the two analytic datasets used in this study. From 36,415 microbiological records retrieved between 2017 and 2022, isolates of non-target species (*n* = 1045) and non-resistant isolates (*n* = 29,912) were excluded (total excluded *n* = 30,957). Dataset A comprised all clinical isolates of the five target species and served as the denominator for the resistance-proportion analyses and the pathogen-specific regression models; these were counted as all isolates of each target species per period. Dataset B comprised the 5458 culture-confirmed MDRO cases (first isolate per species per admission; readmissions separated by ≥30 days counted as separate cases) used for the demographic and clinical analyses, stratified into pre-pandemic (2017–2019; *n* = 1302) and pandemic (2020–2022; *n* = 4156) periods.

Of 36,415 bacterial isolates retrieved across all species during the study period, 30,957 were not eligible for the case-level analysis: isolates not belonging to the five target pathogen groups (*n* = 1045) and non-resistant isolates within the target groups (*n* = 29,912). After retaining only the first isolate per species per admission, 5458 culture-confirmed MDRO cases remained. No cases meeting the MDRO definition were excluded due to incomplete medical records; all 5458 cases had sufficient clinical data for inclusion in the final analysis (Figure 1).

### 2.3. Microbiology and Definitions

All cultures analyzed were clinical cultures ordered as part of routine diagnostic care; surveillance or screening cultures collected solely to detect colonization were not part of the laboratory workflow during the study period. Because clinical infection could not be adjudicated against standardized criteria at the isolate level in this retrospective design, the units of analysis are described throughout as culture-confirmed MDRO cases or clinical isolates rather than as confirmed infections. Pathogen identification and antimicrobial susceptibility testing (AST) were performed using the VITEK 2 automated system (bioMérieux, Marcy-l’Étoile, France). AST results were interpreted according to the Clinical and Laboratory Standards Institute (CLSI) Performance Standards for Antimicrobial Susceptibility Testing (M100) [31,32,33,34,35,36], applying the edition in use during each respective study year (M100-Ed27 through M100-Ed32, 2017–2022). Multidrug-resistant status followed the international consensus definition: acquired non-susceptibility to at least one agent in three or more antimicrobial categories [37]. The target MDROs comprised Carbapenem-resistant *Enterobacterales* (CRE), Carbapenem-resistant *Acinetobacter baumannii* (CRAB), Carbapenem-resistant *Pseudomonas aeruginosa* (CRPA), Methicillin-resistant *Staphylococcus aureus* (MRSA), and Vancomycin-resistant enterococci (VRE). CRE, CRAB, and CRPA were defined as isolates resistant or non-susceptible to at least one carbapenem agent according to CLSI breakpoints. MRSA was defined based on oxacillin or cefoxitin resistance. VRE was defined as *Enterococcus* isolates resistant to vancomycin. The Magiorakos et al. criteria [37] are cited as the general framework for defining multidrug resistance; however, classification in this study was based on the resistance phenotype used for routine surveillance rather than on the full multidrug-resistant definition. Specifically, CRE, CRAB, and CRPA were identified by carbapenem non-susceptibility alone, and MRSA and VRE by methicillin and vancomycin resistance, respectively; isolates were not required to meet the complete Magiorakos definition of non-susceptibility to at least one agent in three or more antimicrobial categories. These carbapenem- and marker-based phenotypes were used because they correspond to the organisms of primary public-health and infection-control concern and are the categories captured consistently in the laboratory information system over the study period.

Infection acquisition was classified using CDC/NHSN-based timing criteria. A hospital-acquired infection (HAI) was defined as an isolate recovered >48 h after hospital admission that was not present or incubating at the time of arrival [38]. Isolates identified within the first 48 h of admission were classified as community-acquired [38,39].

### 2.4. Data Sources and Variables

Comprehensive clinical and demographic data were extracted from the hospital electronic medical record system and laboratory information system. Key variables included age, sex, primary diagnosis, comorbidity status, defined as the presence versus absence of any chronic underlying disease contributing to the Charlson Comorbidity Index [40] and analyzed as a binary variable (present vs. absent), ward type (ICU vs. non-ICU), infection acquisition status, and healthcare utilization measures. To reduce immortal time bias, length of hospital stay was measured from the date of admission to the date of the first positive culture (time at risk), rather than total length of stay. For the regression models, primary diagnosis was entered as a binary variable contrasting urinary tract infection or pneumonia with all other primary diagnoses (reference category). These two syndromes were the most frequent infection-related diagnoses and the presentations most commonly linked to the target Gram-negative pathogens. They were grouped a priori into a single category to preserve degrees of freedom and avoid sparse cells in the pathogen-specific, period-stratified models. The remaining diagnoses were individually infrequent and heterogeneous, and were combined into the reference category. This time-at-risk variable is distinct from the CDC/NHSN timing-based acquisition classification described in Section 2.2 and Section 2.3, and the two were treated as separate variables in all analyses.

### 2.5. Statistical Analysis

Categorical variables were summarized as frequencies and percentages; continuous variables were summarized as means and standard deviations (SDs), and additionally as medians with interquartile ranges (IQRs) when the distribution was skewed (e.g., length of stay). Baseline characteristics were compared between periods using chi-square tests for categorical variables.

Two analytic datasets were used and are shown in Figure 1. Dataset A comprised all species-level clinical isolates of the five target pathogens and provided the denominators for the resistance-proportion analyses and the pathogen-specific regression models. Dataset B comprised the 5458 culture-confirmed MDRO cases (first isolate per species per admission) and was used for the demographic and clinical analyses. The primary descriptive outcome was the proportion of resistant isolates within each pathogen group in each study period. Proportions were calculated as the number of resistant isolates divided by the total number of isolates of that pathogen group during the corresponding period. Absolute differences in percentage points and odds ratios (ORs) with 95% confidence intervals (CIs) were calculated to compare resistance proportions between the pandemic period (2020–2022; study group) and the pre-pandemic period (2017–2019; reference group). These estimates describe changes in the proportion of resistance among clinical isolates and should not be interpreted as direct measures of incidence in the hospitalized population. This study evaluates changes in pathogen-specific resistance proportions among clinical isolates and does not estimate incidence rates in the hospital population due to lack of denominator data (e.g., patient-days).

For the analyses of associated factor, chi-square tests were used for bivariate comparisons. Pathogen-specific univariate and multivariable logistic regression models were then constructed separately for CRE, CRAB, and CRPA within each study period. In each model the dependent variable was the index pathogen group versus the other culture-confirmed MDRO cases of the same period (a one-versus-other-MDROs comparison); the reference group therefore comprised the remaining MDRO cases rather than carbapenem-susceptible isolates of the same species. Because the comparator was the pool of other MDRO cases, these models identify factors associated with the relative distribution of MDRO types among culture-confirmed MDRO cases, and should not be interpreted as risk factors for acquiring infection or resistance per se. The number of cases contributing to each model corresponds to the period-specific totals shown in Table 1 (pre-pandemic *n* = 1302; pandemic *n* = 4156). Variables entered into the multivariable models included sex, age, comorbidity (any chronic underlying disease, present vs. absent), infection acquisition status, primary diagnosis, ward type, and length of hospital stay prior to culture. The five most frequent primary diagnoses at the study hospital were urinary tract infection (UTI), pneumonia, chronic kidney disease, heart failure, and other diagnoses; because each individual diagnosis accounted for less than 10% of cases, the primary diagnosis was analyzed as a binary variable contrasting UTI or pneumonia with all other diagnoses. UTI and pneumonia were grouped because they were the infection syndromes most commonly associated with the target Gram-negative pathogens, and the remaining diagnoses (chronic kidney disease, heart failure, and other) formed the reference category. This primary-diagnosis variable is distinct from the comorbidity variable described above. The results were reported as crude odds ratios (ORs) and adjusted odds ratios (aORs) with 95% CIs. All analyses were performed using IBM SPSS Statistics for Windows, version 26.0 (IBM Corp., Armonk, NY, USA), and a two-sided *p*-value < 0.05 was considered statistically significant. To assess whether the observed associations were robust to differences in case mix between periods, we performed a sensitivity analysis in which the pathogen-specific models were repeated separately within the ICU and non-ICU strata. The direction and statistical significance of the principal associations were consistent with the main analysis; full results are provided in Supplementary Materials.

## 3. Results

### 3.1. Demographic and Clinical Characteristics

During the study period, 36,415 bacterial isolates were retrieved across all species. Of these, 5458 culture-confirmed MDRO cases met all inclusion criteria and constituted the final study population (Figure 1). Demographic and clinical characteristics are summarized in Table 1.

The pre-pandemic period (2017–2019) comprised 1302 cases (23.9%), whereas the pandemic period (2020–2022) comprised 4156 cases (76.1%). The substantially larger number of cases during the pandemic period likely reflects both increased hospital admissions during COVID-19 surges and expanded microbiological culture practices to evaluate suspected secondary bacterial infections in COVID-19 patients.

The overall cohort had a mean age of 63.07 ± 15.84 years, and 64.3% of cases were male. Most cases were aged 60 years or older (62.9%), admitted to non-ICU wards (73.9%), and had a hospital stay of ≤14 days prior to culture (58.5%). Across the full study period, hospital-acquired infection accounted for 53.4% of cases and community-acquired infection for 46.6%.

Several baseline characteristics differed significantly between the two periods (Table 1). During the pandemic period, the proportion of male cases increased from 60.68% to 65.42% (+4.74 percentage points (pp); *p* = 0.002), and the proportion of patients aged ≥60 years rose from 60.29% to 63.74% (+3.45 pp; *p* = 0.025). The proportion of cases with a documented comorbidity decreased from 18.82% to 11.16% (–7.66 pp; *p* < 0.001). The proportion of cases classified as hospital-acquired increased markedly from 40.71% to 57.41% (+16.70 pp; *p* < 0.001), and ICU admissions increased from 19.74% to 28.10% (+8.36 pp; *p* < 0.001). The proportion of cases with a hospital stay > 14 days prior to culture did not differ significantly between periods (42.63% vs. 41.17%; *p* = 0.352), despite a decline in mean length of stay from 23.01 ± 41.30 days to 17.72 ± 21.38 days. Because the distribution of length of stay was right-skewed (standard deviation exceeding the mean), the median (IQR) is also reported and is more representative: 11 (4–28) days pre-pandemic and 11 (5–23) days during the pandemic.

### 3.2. Temporal Trends in the Proportion of Multidrug-Resistant Organisms (2017–2022)

**Figure 2:**
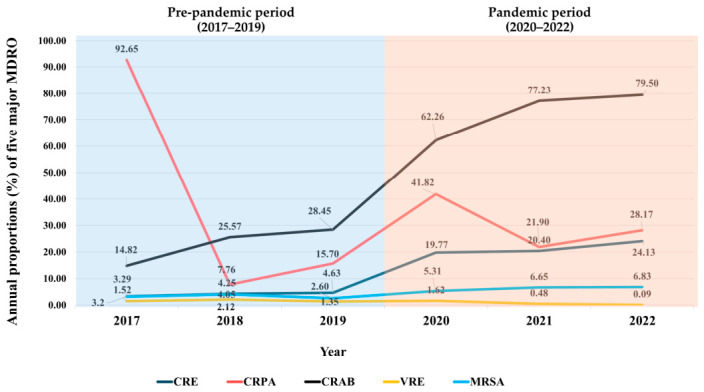
Temporal trends in the annual pathogen-specific proportions of five major MDROs (CRE, CRPA, CRAB, VRE, and MRSA), 2017–2022. Blue shaded area: pre-pandemic period (2017–2019); pink shaded area: pandemic period (2020–2022). Values for each pathogen are proportions within that pathogen group, calculated as the number of resistant isolates divided by the total isolates of that species retrieved in that year, not proportions of all isolates. The annual denominators (total isolates of each species per year) were as follows. *Enterobacterales*: 3467 (2017), 3928 (2018), 3153 (2019), 1416 (2020), 2882 (2021), 2433 (2022). *P. aeruginosa*: 68, 953, 669, 275, 872, 529. *A. baumannii*: 749, 794, 696, 363, 1449, 1010. Enterococci: 1317, 1178, 1037, 925, 1039, 1066. *S. aureus*: 530, 666, 692, 339, 421, 454.

Temporal trends in pathogen-specific proportions of major MDROs are presented in Figure 2. Overall, the proportions of carbapenem-resistant Gram-negative pathogens within their respective pathogen groups increased substantially during the pandemic period, whereas Gram-positive resistance patterns remained stable or declined. These estimates represent proportions within each pathogen group and should not be interpreted as incidence rates in the hospitalized population. Among all pathogens, CRAB showed the most pronounced changes, rising from a relatively low and stable pre-pandemic proportion to a high and sustained level during the pandemic (Figure 2). CRPA demonstrated a markedly different temporal pattern: an exceptionally high proportion in 2017 followed by a sharp decline and subsequent fluctuation; the 2017 value likely reflects the small denominator for that year (68 total *P. aeruginosa* isolates) and should be interpreted with caution. During the pandemic period, CRPA proportions increased relative to the pre-pandemic period despite year-to-year fluctuation. CRE demonstrated a gradual pre-pandemic rise followed by a substantial acceleration during the pandemic, representing an approximately 7.3-fold increase relative to the pre-pandemic mean. VRE exhibited a sustained decline to near-complete disappearance among clinical isolates by the end of the study period, whereas MRSA showed a higher pandemic proportion without a consistent directional trend on annual examination (Figure 2; annual values and denominators are provided in the figure legend).

The between-period distribution of MDROs differed markedly (Table 2). CRAB showed the most pronounced change, rising from 22.87% to 76.11% (OR 10.75), a more than tenfold increase in odds. CRE rose from 4.05% to 21.61% (OR 6.54) and CRPA from 14.32% to 27.15% (OR 2.23). Among Gram-positive pathogens, MRSA increased from 3.28% to 6.34% (OR 2.01), whereas VRE declined from 1.67% to 0.69% (OR 0.41); all between-period differences were significant (*p* < 0.001; full counts, confidence intervals, and absolute changes are given in Table 2). Overall, these findings indicate that the proportion of carbapenem-resistant Gram-negative pathogens among clinical isolates was substantially higher during the pandemic period, while Gram-positive resistance patterns remained relatively stable or declined.

### 3.3. Factors Associated with the Relative Distribution of Pathogen-Specific MDRO Types

Multivariable logistic regression analysis identified distinct and evolving patterns of factors associated with the relative distribution of pathogen-specific MDRO types across the pre-pandemic (2017–2019) and pandemic (2020–2022) periods (Table 3).

#### 3.3.1. Pre-Pandemic Period (2017–2019)

During the pre-pandemic period, male sex was independently associated with lower odds of CRE compared with female sex (aOR 0.51, 95% CI 0.39–0.65; *p* < 0.001), but with higher odds of CRAB (aOR 1.57, 95% CI 1.23–2.02; *p* < 0.001). No significant association between male sex and CRPA was observed. ICU admission was associated with lower odds of CRE (aOR 0.53, 95% CI 0.38–0.75; *p* < 0.001) but higher odds of CRAB (aOR 1.89, 95% CI 1.41–2.56; *p* < 0.001) relative to non-ICU admission. Hospital acquisition was independently associated with higher odds of CRE (aOR 1.42, 95% CI 1.11–1.81; *p* = 0.001). Age, primary diagnosis, comorbidity status, and length of hospital stay prior to culture were not significantly associated with any pathogen-specific outcome during the pre-pandemic period.

#### 3.3.2. Pandemic Period (2020–2022)

During the pandemic period, male sex remained associated with lower odds of CRE (aOR 0.78, 95% CI 0.68–0.89; *p* < 0.001) and was newly associated with higher odds of CRPA (aOR 1.37, 95% CI 1.11–1.72; *p* < 0.01), while no significant association with CRAB was observed (aOR 1.12, 95% CI 0.97–1.27; *p* > 0.05). ICU admission was independently associated with significantly lower odds of CRE (aOR 0.52, 95% CI 0.44–0.61; *p* < 0.001) and CRPA (aOR 0.63, 95% CI 0.49–0.79; *p* < 0.001) compared with non-ICU admission, whereas ICU admission was associated with higher odds of CRAB (aOR 2.13, 95% CI 1.85–2.50; *p* < 0.001). Hospital acquisition was independently associated with higher odds of CRE (aOR 1.24, 95% CI 1.07–1.42; *p* < 0.01), but lower odds of CRPA (aOR 0.55, 95% CI 0.45–0.67; *p* < 0.001). The absence of a documented comorbidity was associated with lower odds of CRPA (aOR 0.61, 95% CI 0.42–0.89; *p* < 0.05). A shorter hospital stay prior to culture (≤14 days vs. >14 days) was associated with lower odds of CRE (aOR 0.75, 95% CI 0.66–0.86; *p* < 0.001) but higher odds of CRAB (aOR 1.16, 95% CI 1.02–1.33; *p* < 0.05) and CRPA (aOR 1.31, 95% CI 1.07–1.60; *p* < 0.01). Primary diagnosis of UTI or pneumonia and age ≥ 60 years were not significantly associated with any pathogen-specific outcome during the pandemic period, except for a modest association between age ≥ 60 years and lower odds of CRPA (aOR 0.80, 95% CI 0.65–0.97).

Overall, non-ICU ward admission was consistently associated with higher odds of CRE in both periods and of CRPA during the pandemic period, and hospital acquisition was independently associated with CRE across both periods. ICU admission was consistently associated with higher odds of CRAB in both periods. Age ≥ 60 years was not independently associated with any pathogen-specific outcome after multivariable adjustment, apart from a modest association with lower odds of CRPA during the pandemic period.

## 4. Discussion

This six-year retrospective study demonstrated a marked proportional shift toward carbapenem-resistant Gram-negative organisms during the COVID-19 pandemic, with the most prominent finding being a substantial increase in the proportions of CRAB and CRE among cultured clinical isolates. These findings are consistent with reports from other settings documenting pandemic-associated changes in AMR epidemiology, especially among Gram-negative pathogens [15,18,19,21,41,42,43].

The most pronounced finding was the sustained increase in CRAB, which rose from 62.26% in 2020 to 79.50% in 2022 among total *A. baumannii* isolates. This trajectory aligns with Thailand’s third and fourth COVID-19 waves, driven by the Alpha and Delta variants, respectively, which caused the most severe surges in hospitalizations and placed critical care resources under extreme strain [44,45].

During the pandemic, ICU overflows into general wards, prolonged patient stays, and intensified broad-spectrum antibiotic use coincided with the observed increase in CRAB proportions, consistent with reports documenting CRAB outbreaks concurrent with COVID-19 patient surges [19,26,46,47]. Importantly, studies have shown that CRAB incidence may not correlate with carbapenem consumption alone, suggesting that disrupted IPC practices, rather than antibiotic selection pressure alone, may be a primary driver [25]. Because molecular typing data were unavailable, a localized clonal outbreak cannot be excluded as a contributing factor.

Unlike CRAB, CRPA increased more gradually during the pandemic period (OR 2.23, 95% CI 1.87–2.65), suggesting that selective pressures predated the pandemic and may have continued during it. This trajectory may reflect the mechanistic diversity of carbapenem resistance in *P. aeruginosa*, encompassing both intrinsic chromosomal pathways (*Opr*D loss, *Amp*C overproduction, and efflux pump upregulation) and acquired mobile metallo-β-lactamase genes (*IMP*- and *VIM*-type carbapenemases) transmitted via integrons and plasmids under sustained antibiotic pressure [48,49]. The divergent temporal trajectories of CRAB and CRPA may also partly reflect inter-species ecological dynamics. *P. aeruginosa* can suppress the growth of *A. baumannii* through iron competition (using more effective siderophore systems) and by producing pyocyanin-mediated oxidative stress, yet both organisms can coexist in mixed-species biofilms and are frequently co-isolated from hospital environmental niches [50]. As CRAB proportions remained elevated after 2021, concurrent CRPA elevation was observed, though the underlying mechanisms remain unclear without environmental or genomic data.

The significant decline in VRE (OR 0.41, 95% CI 0.25–0.68) is noteworthy and is not easily attributable to universal IPC measures alone. A study from Taiwan similarly found that enhanced mask wearing and hand hygiene during the pandemic did not significantly reduce VRE transmission [51]. The VRE decline in our setting may instead reflect shifts in patient case mix, reduced vancomycin prescribing, or changes in the population predisposed to VRE colonization. The proportion of MRSA among *S. aureus* isolates rose between periods (3.28% to 6.34%), and on annual examination increased from 3.21–4.05% before the pandemic to 5.31–6.83% during it. MRSA showed a statistically significant between-period increase (OR 2.01). Part of this change may be attributable to the smaller total *S. aureus* denominator during the pandemic, but the annual MRSA proportion more than doubled over the study period, so a true increase cannot be excluded and the finding warrants continued monitoring. The maintenance of contact precautions may have helped limit MRSA transmission even under healthcare system strain [21,22]. These pathogen-specific divergences underscore that pandemic-related disruption did not affect all MDROs uniformly, and that organism-specific transmission dynamics must be considered when interpreting AMR trends.

The multivariable analyses revealed distinct and pathogen-specific profiles of associated factors. ICU admission was consistently the higher-risk setting for CRAB in both periods, reflecting the high environmental persistence of *A. baumannii*, the intensive use of invasive devices, and the concentration of severely ill patients requiring broad-spectrum carbapenem therapy in critical care units [46]. Conversely, non-ICU ward admission (i.e., lower odds with ICU admission) was associated with higher odds of CRE in both periods, whereas the corresponding association for CRPA reached statistical significance during the pandemic period only and was not significant in the pre-pandemic period. During the pandemic, this pattern extended across CRE and CRPA, consistent with a higher proportion of these carbapenem-resistant isolates being recovered across the broader inpatient environment, possibly related to ICU overflow, the cohorting of COVID-19 patients, and reduced IPC compliance in general wards. The absence of a documented comorbidity was significantly associated with lower odds of CRPA during the pandemic (aOR 0.61, 95% CI 0.42–0.89), consistent with the established predilection of *P. aeruginosa* for patients with chronic lung disease, malignancy, or immunosuppression [48,52]. Although comorbidity status did not reach statistical significance for CRE or CRAB after multivariable adjustment, the direction of the point estimates was consistent, and the absence of statistical significance should not be interpreted as absence of a clinically meaningful relationship.

Male sex was independently associated with lower odds of CRE in both periods, a pattern that likely reflects sex-related differences in underlying disease burden and healthcare utilization patterns rather than a direct biological effect [52]. Shorter hospitalization prior to culture (≤14 days) was associated with lower odds of CRE but higher odds of CRAB and CRPA during the pandemic. For CRE, this is consistent with the established role of prolonged healthcare exposure, through cumulative antibiotic selection pressure, cross-transmission, and extended device exposure, in promoting CRE acquisition [53,54,55]. The association of shorter stay with higher odds of CRAB and CRPA may reflect earlier clinical presentation of healthcare-acquired infection in patients already colonized at or shortly after admission, or increased clinical sampling vigilance during the pandemic for these pathogens. Notably, the proportion of cases with a hospital stay > 14 days prior to culture did not differ between periods despite the decline in mean length of stay, suggesting that the between-period differences in resistance proportions are unlikely to be explained by differential healthcare exposure duration alone.

Several important limitations should be acknowledged. First, the analysis was based on clinical isolates without a hospital-wide population denominator (e.g., patient-days), so incidence rates could not be calculated; the findings therefore describe changes in resistance proportions among clinical isolates rather than direct changes in MDRO incidence. Second, because this was a retrospective laboratory- and record-based study, clinical infection could not be reliably distinguished from colonization at the isolate level. Third, changes in testing practices, admission patterns, and case mix during the pandemic, together with the substantially larger pandemic cohort, may have introduced surveillance and selection bias and could have influenced resistance proportions independently of any true change in resistance burden. Although subgroup sensitivity analyses (ICU/non-ICU and hospital-/community-acquired) were performed to address this, residual compositional effects cannot be excluded. Fourth, a complete history of prior admissions and healthcare exposure was not consistently available and could not be incorporated. Fifth, residual confounding is likely because data on antibiotic consumption, invasive device use, severity of illness, and ward-level IPC conditions were unavailable. Sixth, the absence of molecular typing precluded differentiation between clonal transmission and independent resistance emergence. Finally, as a single-center study at a provincial tertiary referral hospital, generalizability to other settings may be limited.

## 5. Conclusions

The COVID-19 pandemic period was associated with a substantial proportional shift toward carbapenem-resistant Gram-negative pathogens, particularly CRAB and CRE, among clinical isolates in a provincial tertiary care hospital in Thailand. In multivariable analyses, ICU admission was the primary setting associated with CRAB across both periods, while non-ICU admission was associated with higher odds of CRE in both periods and CRPA during the pandemic period, indicating distinct epidemiological niches for these pathogens. Hospital acquisition was associated with CRE in both periods. Several associated factors were observed during the pandemic period: non-ICU admission was associated with higher odds of CRE and CRPA while CRAB was associated with ICU admission, and shorter hospitalization prior to culture was associated with higher odds of CRAB and CRPA while longer stay was associated with CRE. These findings support strengthening pathogen-specific antimicrobial stewardship, surveillance, and infection prevention strategies across all inpatient settings—not only within ICUs—during periods of healthcare system disruption.

## Figures and Tables

**Table 1:** Demographic and Clinical Characteristics of Study Population.

Characteristic	Pre-Pandemic (2017–2019) *n* = 1302	Pandemic (2020–2022) *n* = 4156	% Change (Pandemic vs. Pre-Pandemic)	Total *n* = 5458	*p*-Value
Sex, *n* (%)					0.002
Male	790 (60.68)	2719 (65.42)	+4.74 pp	3509 (64.29)	
Female	512 (39.32)	1437 (34.58)	−4.74 pp	1949 (35.71)	
Age (years)					0.025
Mean ± SD	61.89 ± 16.41	63.44 ± 15.65		63.07 ± 15.84	
<60 years, *n* (%)	517 (39.71)	1507 (36.26)	−3.45 pp	2024 (37.08)	
≥60 years, *n* (%)	785 (60.29)	2649 (63.74)	+3.45 pp	3434 (62.92)	
Comorbidity/Underlying disease, *n* (%)					<0.001
Present	245 (18.82)	464 (11.16)	−7.66 pp	709 (12.99)	
Absent	1057 (81.18)	3692 (88.84)	+7.66 pp	4749 (87.01)	
Type of Infection, *n* (%)					<0.001
Community-acquired	772 (59.29)	1770 (42.59)	−16.70 pp	2542 (46.57)	
Hospital-acquired	530 (40.71)	2386 (57.41)	+16.70 pp	2916 (53.43)	
Ward Type, *n* (%)					<0.001
General ward	1045 (80.26)	2988 (71.90)	−8.36 pp	4033 (73.87)	
ICU	257 (19.74)	1168 (28.10)	+8.36 pp	1425 (26.13)	
Length of Hospital Stay (time from admission to first positive culture), *n* (%)					0.352
≤14 days	747 (57.37)	2445 (58.83)	+1.46 pp	3192 (58.48)	
>14 days	555 (42.63)	1711 (41.17)	−1.46 pp	2266 (41.52)	
Mean ± SD (days)	23.01 ± 41.30	17.72 ± 21.38		18.98 ± 27.56	
Median (IQR) (days)	11 (4–28)	11 (5–23)		11 (4–24)	

SD: Standard Deviation; IQR: interquartile range.

**Table 2:** Proportions and Odds Ratios of Multidrug-Resistant Organisms Before and During COVID-19 Pandemic.

MDRO	Pre-Pandemic (2017–2019) *n*/*N* (%)	Pandemic (2020–2022) *n*/*N* (%)	Absolute Change (Percentage Points)	Odds Ratio (OR)	95% CI	*p*-Value
CRAB	512/2239 (22.87)	2148/2822 (76.11)	+53.24	10.75	9.43–12.26	<0.001
CRE	427/10,548 (4.05)	1455/6731 (21.61)	+17.56	6.54	5.84–7.32	<0.001
CRPA	242/1690 (14.32)	455/1676 (27.15)	+12.83	2.23	1.87–2.65	<0.001
MRSA	62/1888 (3.28)	77/1214 (6.34)	+3.08	2.01	1.42–2.83	<0.001
VRE	59/3532 (1.67)	21/3030 (0.69)	−0.98	0.41	0.25–0.68	<0.001

Abbreviations: CRE, carbapenem-resistant *Enterobacterales*; CRPA, carbapenem-resistant *Pseudomonas aeruginosa*; CRAB, carbapenem-resistant *Acinetobacter baumannii*; VRE, vancomycin-resistant Enterococcus; MRSA, methicillin-resistant *Staphylococcus aureus*. For each pathogen, the denominator (N) is the total number of clinical isolates of that species recovered during the period, regardless of resistance status (Dataset A in Figure 1); the numerator (*n*) is the number of carbapenem-resistant (CRE, CRPA, CRAB) or phenotypically resistant (MRSA, VRE) isolates.

**Table 3:** Multivariable Analysis of Factors Associated with the Relative Distribution of Pathogen-Specific MDRO Types (each pathogen group vs. the other MDRO cases) in the Pre-Pandemic (2017–2019) and Pandemic (2020–2022) Periods.

Factor	CRE aOR (95% CI)		CRAB aOR (95% CI)		CRPA aOR (95% CI)	
	Pre-Pandemic (2017–2019)	Pandemic (2020–2022)	Pre-Pandemic (2017–2019)	Pandemic (2020–2022)	Pre-Pandemic (2017–2019)	Pandemic (2020–2022)
Male	0.51 (0.39–0.65) ***	0.78 (0.68–0.89) ***	1.57 (1.23–2.02) ***	1.12 (0.97–1.27)	1.35 (0.99–1.83)	1.37 (1.11–1.72) **
Age ≥ 60 years	1.27 (0.99–1.64)	0.98 (0.86–1.14)	0.90 (0.71–1.15)	1.11 (0.98–1.27)	0.84 (0.63–1.12)	0.80 (0.65–0.97) *
Infectious disease primary diagnosis (UTI or pneumonia)	0.91 (0.63–1.31)	0.89 (0.73–1.10)	1.32 (0.93–1.88)	0.98 (0.81–1.20)	0.74 (0.46–1.18)	1.31 (0.98–1.74)
Comorbidity/ Underlying disease	0.99 (0.72–1.36)	1.04 (0.85–1.28)	1.12 (0.83–1.51)	1.13 (0.93–1.39)	0.86 (0.59–1.25)	0.61 (0.42–0.89) *
Hospital infection	1.42 (1.11–1.81) **	1.24 (1.07–1.42) **	0.86 (0.68–1.09)	1.05 (0.92–1.19)	0.77 (0.57–1.03)	0.55 (0.45–0.67) ***
ICU admission	0.53 (0.38–0.75) ***	0.52 (0.44–0.61) ***	1.89 (1.41–2.56) ***	2.13 (1.85–2.50) ***	0.84 (0.58–1.20)	0.63 (0.49–0.79) ***
Length of stay ≤ 14 days	0.90 (0.70–1.16)	0.75 (0.66–0.86) ***	0.99 (0.78–1.27)	1.16 (1.02–1.33) *	1.16 (0.87–1.55)	1.31 (1.07–1.60) **

* *p* < 0.05; ** *p* < 0.01; *** *p* < 0.001. aOR = adjusted odds ratio; CI = confidence interval. CRE = Carbapenem-Resistant *Enterobacterales*; CRAB = Carbapenem-Resistant *Acinetobacter baumannii*; CRPA = Carbapenem-Resistant *Pseudomonas aeruginosa*. Reference categories: female sex; age < 60 years; presence of comorbidity; non-infectious-disease primary diagnosis (chronic kidney disease, heart failure, or other); community-acquired infection; non-ICU admission; length of stay > 14 days.

## Data Availability

The original contributions presented in this study are included in the article/Supplementary Materials. Further inquiries can be directed to the corresponding author.

## Supplementary Materials

The following supporting information can be downloaded at: https://www.mdpi.com/article/10.3390/medsci14030366/s1, Table S1: Demographic characteristics of the study population stratified by year (2017–2022). Table S2: Factors associated with carbapenem-resistant Enterobacterales (CRE) before the COVID-19 pandemic (logistic regression analysis). Table S3: Factors associated with carbapenem-resistant Enterobacterales (CRE) during the COVID-19 pandemic (logistic regression analysis). Table S4: Factors associated with carbapenem-resistant Acinetobacter baumannii (CRAB) before the COVID-19 pandemic (logistic regression analysis). Table S5: Factors associated with carbapenem-resistant Acinetobacter baumannii (CRAB) during the COVID-19 pandemic (logistic regression analysis). Table S6: Factors associated with carbapenem-resistant Pseudomonas aeruginosa (CRPA) before the COVID-19 pandemic (logistic regression analysis). Table S7: Factors associated with carbapenem-resistant Pseudomonas aeruginosa (CRPA) during the COVID-19 pandemic (logistic regression analysis). Table S8: Sensitivity analysis of factors associated with the relative distribution of MDRO types, stratified by ward type (ICU versus non-ICU).
