# Supplementary material for: Changes in the Epidemiology of Multidrug-Resistant Organisms During the COVID-19 Pandemic: A Six-Year Retrospective Study at a Tertiary Care Hospital in Northeastern Thailand
Source: Med Sci (Basel). 2026 Jul 1;14(3):366. doi: 10.3390/medsci14030366 (PMC13414112; doi:10.3390/medsci14030366)
Supplement: Supplementary file 1 [file medsci-14-00366-s001.zip › medsci-4321028-supplementary.pdf]

Supplementary Data and Revised Tables

1. Demographic characteristics by individual year

This table presents each variable disaggregated by year, with the corresponding frequency and percentage.

Table S1. Demographic characteristics (n = 5,458), stratified by year.

Variable / Period	2017<br>n = 325	2018<br>n = 496	2019<br>n = 481	2020<br>n = 654	2021<br>n = 1,931	2022<br>n = 1,571
Sex						
Male	192 (59.08)	317 (63.91)	281 (58.42)	419 (64.07)	1,301 (67.37)	999 (63.59)
Female	133 (40.92)	179 (36.09)	200 (41.58)	235 (35.93)	630 (32.63)	572 (36.41)
Age (years)						
16–21	6 (1.85)	7 (1.41)	13 (2.70)	12 (1.83)	19 (0.98)	12 (0.76)
22–34	9 (2.77)	26 (5.24)	23 (4.78)	31 (4.74)	96 (4.97)	54 (3.44)
35–44	35 (10.77)	48 (9.68)	36 (7.48)	49 (7.49)	146 (7.56)	98 (6.24)
45–59	68 (20.92)	127 (25.60)	119 (24.74)	165 (25.23)	455 (23.56)	370 (23.55)
≥60	207 (63.69)	288 (58.06)	290 (60.29)	397 (60.70)	1,215 (62.92)	1,037 (66.01)
Mean (SD)	62.54 (15.85)	61.34 (16.21)	61.95 (16.98)	62.35 (16.05)	63.12 (15.92)	64.30 (15.11)
Min–Max	16–98	17–96	16–96	16–99	16–99	17–99
Primary diagnosis (top 5)						
Pneumonia	34 (10.46)	56 (11.29)	43 (8.94)	50 (7.65)	182 (9.43)	116 (7.38)
CKD stage 5	9 (2.77)	17 (3.43)	16 (3.33)	35 (5.35)	83 (4.30)	69 (4.39)
UTI	2 (0.62)	20 (4.03)	15 (3.12)	39 (5.96)	33 (1.71)	54 (3.44)
Heart failure	2 (0.62)	11 (2.22)	14 (2.91)	18 (2.75)	63 (3.26)	52 (3.31)
Brain death	8 (2.46)	11 (2.22)	9 (1.87)	14 (2.14)	68 (3.52)	41 (2.61)
Other	270 (83.08)	381 (76.81)	384 (79.83)	498 (76.15)	1,502 (77.78)	1,239 (78.87)
Comorbidity						
Absent	256 (78.77)	406 (81.85)	395 (82.12)	650 (99.39)	1,670 (86.48)	1,372 (87.33)
Present	69 (21.23)	90 (18.15)	86 (17.88)	4 (0.61)	261 (13.52)	199 (12.67)
Type of infection						
Community-acquired	209 (64.31)	309 (62.30)	254 (52.81)	289 (44.19)	790 (40.91)	691 (43.98)
Nosocomial	116 (35.69)	187 (37.70)	227 (47.19)	365 (55.81)	1,141 (59.09)	880 (56.02)
Ward type						
ICU	62 (19.08)	97 (19.56)	98 (20.37)	127 (19.42)	552 (28.59)	489 (31.13)
Non-ICU	263 (80.92)	399 (80.44)	383 (79.63)	527 (80.58)	1,379 (71.41)	1,082 (68.87)
Length of hospital stay						
<7 days	126 (38.77)	171 (34.48)	173 (35.97)	247 (37.77)	639 (33.09)	550 (35.01)
7–14 days	68 (20.92)	105 (21.17)	104 (21.62)	149 (22.78)	493 (25.53)	367 (23.36)
>14 days	131 (40.31)	220 (44.35)	204 (42.41)	258 (39.45)	799 (41.38)	654 (41.63)
Median (IQR)	9.00 (22.50)	12.00 (23.00)	11.00 (25.00)	10.00 (19.00)	11.00 (18.00)	11.00 (20.00)
Min–Max	1–378	1–378	1–923	1–270	1–198	1–265

SD, standard deviation; IQR, interquartile range; CKD, chronic kidney disease; UTI, urinary tract infection; ICU, intensive care unit.

2. Comparison of resistant vs. susceptible isolates before and during the pandemic

Table S2. Factors associated with carbapenem-resistant *Enterobacterale*s (CRE) before the COVID-19 pandemic (logistic regression analysis).

Factor	CRE<br>n (%)	Non-CRE<br>n (%)	Crude OR<br>(95% CI)	Adjusted OR<br>(95% CI)	p-value
Sex					**<0.001**
Male	217 (29.77)	512 (70.23)	1	1	
Female	210 (46.46)	242 (53.54)	0.49 (0.38–0.52) [verify]	0.51 (0.39–0.65)	
Age					**0.062**
<60	148 (31.42)	323 (68.58)	1	1	
≥60	279 (39.30)	431 (60.70)	1.41 (1.10–1.81)	1.27 (0.99–1.64)	
Primary diagnosis (top 5)					**0.604**
CKD5/HF/Brain death/Other	373 (36.25)	656 (63.75)	1	1	
Pneumonia/UTI	54 (35.53)	98 (64.47)	0.97 (0.98–1.38) [verify]	0.91 (0.63–1.31)	

Factor	CRE n (%)	Non-CRE n (%)	Crude OR (95% CI)	Adjusted OR (95% CI)	p-value
Comorbidity<br>Absent<br>Present	 346 (35.97)<br>81 (36.99)	 616 (64.03)<br>138 (63.01)	 1<br>1.04 (0.77–1.42)	 1<br>0.99 (0.72–1.36)	**0.957**
Type of infection<br>Community-acquired<br>Nosocomial	 231 (31.98)<br>214 (41.55)	 453 (68.02)<br>301 (58.45)	 1<br>1.51 (1.19–1.92)	 1<br>1.42 (1.11–1.81)	**0.005**
Length of hospital stay<br>>14 days<br>≤14 days	 178 (33.84)<br>249 (38.02)	 348 (66.16)<br>406 (61.98)	 1<br>1.20 (0.94–1.52)	 1<br>0.90 (0.70–1.16)	**0.403**

Table S3. Factors associated with carbapenem-resistant *Enterobacterales* (CRE) during the COVID-19 pandemic (logistic regression analysis).

Factor	CRE n (%)	Non-CRE n (%)	Crude OR (95% CI)	Adjusted OR (95% CI)	p-value
Sex<br>Female<br>Male	 560 (39.97)<br>895 (33.68)	 841 (60.03)<br>1,762 (66.32)	 1<br>0.76 (0.67–0.87)	 1<br>0.78 (0.68–0.89)	**<0.001**
Age<br><60<br>≥60	 515 (35.25)<br>940 (36.20)	 946 (64.75)<br>1,657 (63.80)	 1<br>1.04 (0.91–1.19)	 1<br>0.98 (0.86–1.14)	**0.881**
Primary diagnosis (top 5)<br>CKD5/HF/Brain death/Other<br>Pneumonia/UTI	 1,292 (35.97)<br>163 (34.98)	 2,300 (64.03)<br>303 (65.02)	 1<br>0.96 (0.78–1.17)	 1<br>0.89 (0.73–1.10)	**0.287**
Comorbidity<br>Absent<br>Present	 1,284 (35.59)<br>171 (38.00)	 2,324 (64.41)<br>279 (62.00)	 1<br>1.11 (0.91–1.36)	 1<br>1.04 (0.86–1.28)	**0.680**
Type of infection<br>Community-acquired<br>Nosocomial	 561 (33.31)<br>894 (37.66)	 1,123 (66.69)<br>1,480 (62.34)	 1<br>1.21 (1.06–1.38)	 1<br>1.24 (1.07–1.42)	**0.001**
Length of hospital stay<br>>14 days<br>≤14 days	 910 (39.88)<br>545 (30.69)	 1,372 (60.12)<br>1,231 (69.31)	 1<br>0.67 (0.58–0.76)	 1<br>0.75 (0.66–0.86)	**<0.001**

Table S4. Factors associated with carbapenem-resistant *Acinetobacter baumannii* (CRAB) before the COVID-19 pandemic (logistic regression analysis).

Factor	CRAB n (%)	Non-CRAB n (%)	Crude OR (95% CI)	Adjusted OR (95% CI)	p-value
Sex<br>Female<br>Male	 165 (36.50)<br>347 (47.60)	 287 (63.50)<br>382 (52.40)	 1<br>1.58 (1.24–2.01)	 1<br>1.57 (1.23–2.02)	**<0.001**
Age<br><60<br>≥60	 215 (45.65)<br>297 (41.83)	 256 (54.35)<br>413 (58.17)	 1<br>0.86 (0.68–1.08)	 1<br>0.90 (0.71–1.15)	**0.418**
Primary diagnosis (top 5)<br>CKD5/HF/Brain death/Other<br>Pneumonia/UTI	 438 (42.57)<br>74 (48.68)	 591 (57.43)<br>78 (51.32)	 1<br>1.28 (0.91–1.80)	 1<br>1.32 (0.93–1.88)	**0.123**
Comorbidity<br>Absent<br>Present	 414 (43.04)<br>98 (44.75)	 548 (56.96)<br>121 (55.25)	 1<br>1.07 (0.80–1.44)	 1<br>1.12 (0.83–1.51)	**0.464**
Type of infection<br>Community-acquired<br>Nosocomial	 304 (45.65)<br>208 (40.39)	 362 (54.35)<br>307 (59.61)	 1<br>1.23 (0.98–1.56)	 1<br>0.86 (0.68–1.09)	**0.224**
Length of hospital stay<br>>14 days<br>≤14 days	 277 (42.29)<br>235 (44.68)	 378 (57.71)<br>291 (55.32)	 1<br>1.10 (0.87–1.39)	 1<br>0.99 (0.78–1.27)	**0.982**

Table S5. Factors associated with carbapenem-resistant *Acinetobacter baumannii* (CRAB) during the COVID-19 pandemic (logistic regression analysis).

Factor	CRAB n (%)	Non-CRAB n (%)	Crude OR (95% CI)	Adjusted OR (95% CI)	p-value
Sex					**0.107**
Female	714 (50.96)	687 (49.04)	1	1	
Male	1,434 (53.97)	1,223 (46.03)	1.13 (0.99–1.28)	1.12 (0.97–1.27)	
Age					**0.113**
<60	760 (52.02)	701 (47.98)	1	1	
≥60	1,338 (53.45)	1,209 (46.55)	1.06 (0.93–1.20)	1.11 (0.98–1.27)	
Primary diagnosis (top 5)					**0.912**
CKD5/HF/Brain death/Other	1,908 (53.12)	1,684 (46.88)	1	1	
Pneumonia/UTI	240 (51.50)	226 (48.50)	0.94 (0.77–1.14)	0.98 (0.81–1.20)	
Comorbidity					**0.219**
Absent	1,902 (52.72)	1,706 (47.28)	1	1	
Present	246 (54.67)	204 (45.33)	1.08 (0.89–1.32)	1.13 (0.93–1.39)	
Type of infection					**0.455**
Community-acquired	877 (52.08)	807 (47.92)	1	1	
Nosocomial	1,271 (53.54)	1,103 (46.46)	1.06 (0.94–1.20)	1.05 (0.92–1.19)	
Length of hospital stay					**0.022**
>14 days	1,134 (49.69)	1,148 (50.31)	1	1	
≤14 days	1,014 (57.09)	762 (42.91)	1.35 (1.19–1.53)	1.16 (1.02–1.33)	

Table S6. Factors associated with carbapenem-resistant *Pseudomonas aeruginosa* (CRPA) before the COVID-19 pandemic (logistic regression analysis).

Factor	CRPA n (%)	Non-CRPA n (%)	Crude OR (95% CI)	Adjusted OR (95% CI)	p-value
Sex					**0.060**
Female	77 (17.04)	375 (82.96)	1	1	
Male	165 (22.63)	564 (77.37)	1.42 (1.06–1.92)	1.35 (0.99–1.83)	
Age					**0.229**
<60	108 (22.93)	363 (77.07)	1	1	
≥60	134 (18.87)	576 (81.13)	0.78 (0.59–1.04)	0.84 (0.63–1.12)	
Primary diagnosis (top 5)					**0.204**
CKD5/HF/Brain death/Other	218 (21.19)	811 (78.81)	1	1	
Pneumonia/UTI	24 (15.79)	128 (84.21)	0.70 (0.44–1.11)	0.74 (0.46–1.18)	
Comorbidity					**0.426**
Absent	202 (21.00)	760 (79.00)	1	1	
Present	40 (18.26)	179 (81.74)	0.84 (0.58–1.22)	0.86 (0.59–1.25)	
Type of infection					**0.078**
Community-acquired	149 (22.37)	517 (77.63)	1	1	
Nosocomial	93 (18.06)	422 (81.94)	0.76 (0.57–1.02)	0.77 (0.57–1.03)	
Length of hospital stay					**0.316**
>14 days	129 (19.69)	526 (80.31)	1	1	
≤14 days	113 (21.48)	413 (78.52)	1.12 (0.84–1.48)	1.16 (0.87–1.55)	

Table S7. Factors associated with carbapenem-resistant Pseudomonas aeruginosa (CRPA) during the COVID-19 pandemic (logistic regression analysis).

Factor	CRPA n (%)	Non-CRPA n (%)	Crude OR (95% CI)	Adjusted OR (95% CI)	p-value
Sex					**0.004**
Female	127 (9.06)	1,274 (90.94)	1	1	
Male	328 (12.34)	2,329 (87.66)	1.41 (1.14–1.75)	1.37 (1.11–1.72)	
Age					**0.027**
<60	186 (12.73)	1,275 (87.27)	1	1	

Factor	CRPA<br>n (%)	Non-CRPA<br>n (%)	Crude OR<br>(95% CI)	Adjusted OR<br>(95% CI)	p-value
≥60	269 (10.36)	2,328 (89.64)	0.79 (0.65–0.97)	0.80 (0.65–0.97)	
Primary diagnosis (top 5)<br>CKD5/HF/Brain death/Other<br>Pneumonia/UTI	 392 (10.91)<br>63 (13.52)	 3,200 (89.09)<br>403 (86.48)	 1<br>1.28 (0.96–1.70)	 1<br>1.31 (0.98–1.74)	**0.072**
Comorbidity<br>Absent<br>Present	 422 (11.70)<br>33 (7.33)	 3,186 (88.30)<br>417 (92.67)	 1<br>0.60 (0.41–0.86)	 1<br>0.61 (0.42–0.89)	**0.010**
Type of infection<br>Community-acquired<br>Nosocomial	 246 (14.61)<br>209 (8.80)	 1,438 (85.39)<br>2,165 (91.20)	 1<br>0.56 (0.46–0.69)	 1<br>0.55 (0.45–0.67)	**<0.001**
Length of hospital stay<br>>14 days<br>≤14 days	 238 (10.43)<br>217 (12.22)	 2,044 (89.57)<br>1,559 (87.79)	 1<br>1.20 (0.98–1.45)	 1<br>1.31 (1.07–1.60)	**0.009**

OR, odds ratio; CI, confidence interval. Reference categories are indicated by OR = 1. CKD5, chronic kidney disease stage 5; HF, heart failure; UTI, urinary tract infection. Cells marked "[verify]" contain a value in the source document whose point estimate and confidence interval appear inconsistent and should be confirmed against the statistical output.

Table S8. Sensitivity analysis: factors associated with the relative distribution of MDRO types, stratified by ward type (ICU vs. non-ICU)

Each pathogen group was modeled as the index MDRO versus the other MDRO cases (one-vs-others) using multivariable logistic regression, run separately within the ICU and non-ICU strata and within each period. Models were adjusted for sex, age group, hospital acquisition, and length of stay (≤14 vs >14 days). Ward type was the stratifying variable and therefore not entered as a covariate.

Pathogen	Period	Ward stratum	n (events)	Male sex<br>aOR (95% CI)	Age ≥60 y<br>aOR (95% CI)	Hospital acquisition<br>aOR (95% CI)	LOS ≤14 d<br>aOR (95% CI)
**CRE**	Pre-pandemic	ICU	257 (58)	0.62 (0.34–1.12)	1.40 (0.75–2.60)	1.22 (0.65–2.28)	0.86 (0.46–1.60)
	Pre-pandemic	non-ICU	1045 (369)	0.55 (0.42–0.72)***	1.21 (0.92–1.58)	1.83 (1.41–2.37)***	1.30 (0.99–1.70)
	Pandemic	ICU	1168 (285)	0.96 (0.72–1.27)	1.13 (0.86–1.50)	0.86 (0.65–1.12)	1.12 (0.86–1.47)
	Pandemic	non-ICU	2988 (1170)	0.75 (0.64–0.87)***	0.97 (0.83–1.14)	1.52 (1.31–1.77)***	1.40 (1.19–1.64)***
**CRAB**	Pre-pandemic	ICU	257 (134)	1.76 (1.05–2.94)*	0.69 (0.42–1.16)	1.18 (0.69–2.03)	1.42 (0.85–2.39)
	Pre-pandemic	non-ICU	1045 (378)	1.62 (1.24–2.12)***	0.99 (0.76–1.29)	1.04 (0.81–1.35)	0.91 (0.70–1.19)
	Pandemic	ICU	1168 (768)	1.01 (0.78–1.32)	0.99 (0.77–1.28)	1.57 (1.23–2.01)***	1.01 (0.79–1.30)
	Pandemic	non-ICU	2988 (1380)	1.15 (0.99–1.33)	1.19 (1.03–1.39)*	1.02 (0.88–1.18)	0.79 (0.68–0.92)**
**CRPA**	Pre-pandemic	ICU	257 (45)	1.04 (0.53–2.03)	1.09 (0.56–2.11)	0.97 (0.49–1.95)	0.80 (0.40–1.57)
	Pre-pandemic	non-ICU	1045 (197)	1.56 (1.11–2.18)**	0.76 (0.55–1.04)	0.87 (0.63–1.20)	0.87 (0.63–1.19)
	Pandemic	ICU	1168 (100)	1.07 (0.68–1.70)	0.73 (0.48–1.12)	0.53 (0.35–0.80)**	0.68 (0.44–1.05)
	Pandemic	non-ICU	2988 (355)	1.47 (1.15–1.89)**	0.83 (0.66–1.05)	0.60 (0.48–0.75)***	0.74 (0.59–0.93)**

*aOR, adjusted odds ratio; CI, confidence interval; LOS, length of stay; HAI, hospital-acquired infection. * $p < 0.05$; ** $p < 0.01$; *** $p < 0.001$. Reference categories: female sex; age <60 years; community-acquired infection; LOS >14 days. The direction and significance of the principal associations were consistent with the main analysis (Table 3), indicating that the findings are not explained by ward-type composition.*
